# Quantum mechanical electronic and geometric parameters for DNA k-mers as features for machine learning

**DOI:** 10.1038/s41597-024-03772-5

**Published:** 2024-08-22

**Authors:** Kairi Masuda, Adib A. Abdullah, Patrick Pflughaupt, Aleksandr B. Sahakyan

**Affiliations:** MRC WIMM Centre for Computational Biology, MRC Weatherall Institute of Molecular Medicine, Radcliffe Department of Medicine, University of Oxford, Oxford, OX3 9DS UK

**Keywords:** Machine learning, Computational biophysics

## Abstract

We are witnessing a steep increase in model development initiatives in genomics that employ high-end machine learning methodologies. Of particular interest are models that predict certain genomic characteristics based solely on DNA sequence. These models, however, treat the DNA as a mere collection of four, A, T, G and C, letters, dismissing the past advancements in science that can enable the use of more intricate information from nucleic acid sequences. Here, we provide a comprehensive database of quantum mechanical (QM) and geometric features for all the permutations of 7-meric DNA in their representative B, A and Z conformations. The database is generated by employing the applicable high-cost and time-consuming QM methodologies. This can thus make it seamless to associate a wealth of novel molecular features to any DNA sequence, by scanning it with a matching k-meric window and pulling the pre-computed values from our database for further use in modelling. We demonstrate the usefulness of our deposited features through their exclusive use in developing a model for A->C mutation rates.

## Background & Summary

Machine learning techniques are now being actively pursued in all fields, including genomics. The main driver of this phenomenon is the rapid development in computer performance and efficiency as predicted by Moore’s Law^1^. The explosion of digital data necessary to feed the machine learning algorithms has also been a major contributor to the ever-increasing adoption of machine learning. In the field of genomics, big data, especially from high-throughput sequencing technologies, have been utilised to develop many successful machine learning-based models to address various biological problems^2^. Models were built to predict G-quadruplex formation^3^, effective gene expression^4^, splicing events^5,6^, specificities of DNA- and RNA-binding proteins^7^, effects of non-coding variants^8^, epigenomic profiles^9^, transcription factor binding^10^, regulatory code of the accessible genome^11^, DNA methylation^12^, cancer driver genes^13^, and many other biological phenomena.

Most of these works mainly focus on the underlying oligonucleotide letter strings to devise the features for machine learning, requiring a massive amount of data to decipher and exhaust information out of nucleic acid sequences. These sequence-based initiatives, despite being successful, still overlook a decade’s worth of information and advancement accumulated on the inference of physicochemical properties of oligonucleotides in their varying sequence context and structure. Many such properties are commonly calculable from molecular modelling, at coarse and atomistic scales *via* molecular mechanics (MM) and quantum mechanical (QM) electronic techniques. To address this limitation, we propose incorporating features that capture the underlying electronic properties, as most molecular characteristics can be considered as derivatives of, hence, ultimately determined by, the underlying electronic/QM characteristics. Although computationally expensive, electronic, energetic, and structure-based calculations provide highly accurate results of molecular behaviours^14,15^, hence, have the potential to complement standard bioinformatics techniques based on genomic sequence analysis^16^. Thus, by pre-calculating electronic properties for different sequence contexts, we can parameterise and integrate them into the feature generation stage of machine learning models, still keeping the model purely sequence-based in terms of the required primary information.

Electronic properties can explain important DNA behaviours. For example, guanine is susceptible to oxidative damage, which has been associated with its low ionisation potential, facilitating electron loss and oxidation^17,18^. Prior works have also shown that the increased propensity to damage by reactive oxygen species (ROS) at the 5’guanine within guanine-guanine dyads is driven by the reduced ionisation potential^19^. Moreover, the formation of 8-oxo-guanine at Z-DNA sites, one of the three DNA conformations we have calculated the electronic properties on, has been shown to modulate gene regulation through the electronic properties of the nucleobases and their sequence context^20^. As such, incorporating these electronic properties can potentially improve predictive modelling tasks and improve the interpretability of the subsequent model decision-making processes. Hence, this necessitates our work on developing purely sequence-driven electronic properties for standard nucleic acid conformations.

**Fig. 1:**
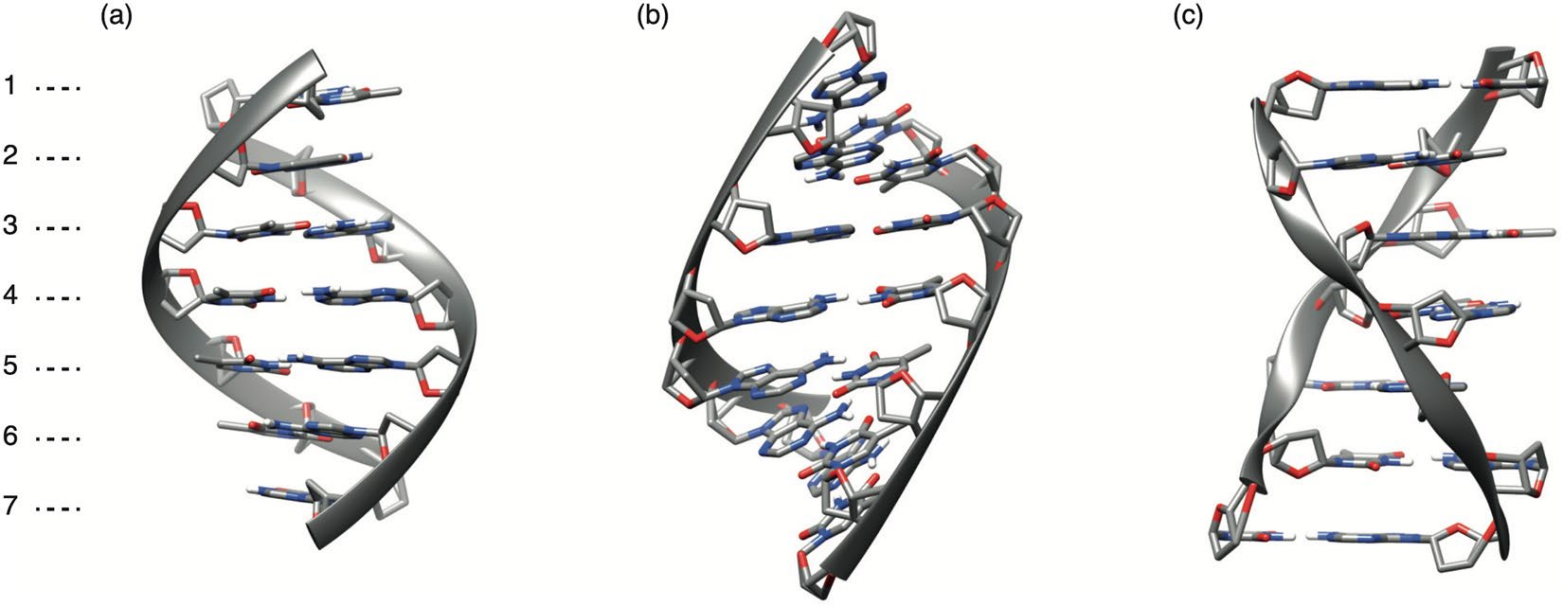
Representative molecular structures of the 7-mer double-stranded DNA models used in this study. The structures are brought for (**a**) B-DNA, (**b**) A-DNA, and (**c**) Z-DNA conformations. For clarity, backbones are represented by ribbons. These structures have seven nucleotide spans at both strands.

To make the above possible, here we performed large-scale semi-empirical QM calculations for all possible DNA heptamers in their three, B, A and Z, representative conformations (Fig. 1). We began with DNA 3D model development and geometry optimisation, followed by semi-empirical calculations. A number of geometric values of the optimised models were also measured and included as part of the dataset. The outcomes of these calculations can be applied as similar values to the machine learning algorithms, where their relevance and information content will be assessed accordingly during the learning process. As a proof of concept, we briefly demonstrated that our DNA heptamer semi-empirical properties along with their geometric measurements were able to predict A to C spontaneous mutation rates^21^ from DNA sequence when they were applied as sole machine learning features with no direct sequence encoding.

We believe our deposited data presented in this work, comprised of 3D geometric and physicochemical properties of 24,576 non-redundant DNA 7-mer duplex structures (not counting the shorter-span 6-mer analogues) in B, A and Z conformations (Fig. 1), will be useful and applicable to enhance the machine learning process in solving DNA-driven biological problems. Such a dataset is unique for varying oligonucleotides, even though databases of many MM and QM properties exist for other types of molecules, many reported in the same, Scientific Data, journal. Examples of other QM-based datasets published in the same spirit are physico-chemical properties of 31,618 electroactive molecules for the development of aqueous redox flow batteries^22^, optimised molecular geometries and thermodynamic data of more than 665,000 biologically and pharmacologically relevant molecules^23^, electronic charge density of crystalline materials from Materials Project database^24^, molecular conformations of 450,000 small- and mid-sized organic molecules^25^, molecular geometries and spectral properties of 61,489 crystal-forming organic molecules^26^, equilibrium conformations for small organic molecules^27^, QM calculations of over 200,000 organic radical species and 40,000 associated closed-shell molecules^28^, all-atom force-field parameters, molecular dynamics trajectories, QM properties, and curated physicochemical descriptors of more than 300 antimicrobial compounds^29^, excited state information of 173,000 organic molecules^30^, conformational energies and geometries of di- and tripeptides^31^, and QM structures and properties of 134,000 small organic molecules^32^.

## Methods

**Fig. 2:**
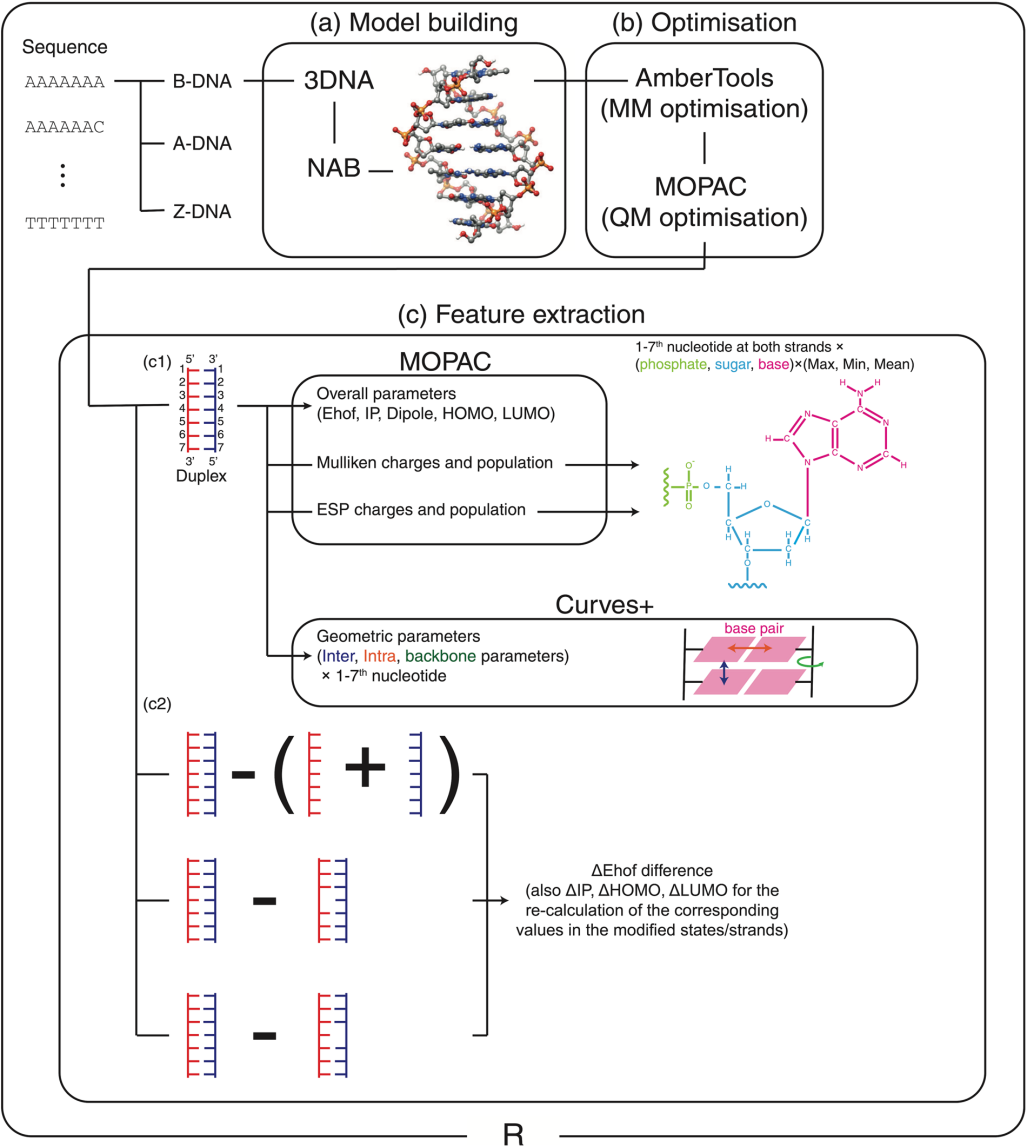
A schematic workflow of the dataset generation used in this study. In the whole procedure, we used R programming language for processing the data. (**a**) To make molecular DNA models, we used web 3DNA and NAB programmes, and a typical resultant DNA model is shown as an illustration. (**b**) Next, we optimised these models in two steps. First *via* MM optimisation by AmberTools21, next *via* QM optimisation by MOPAC2016. (**c1**) After optimisation, we extracted electronic features by single-point MOPAC2016 calculations, and geometric features by using Curves+. For charges and populations, we calculated the maximum, minimum, and mean values of phosphate, sugar, and base moieties. (**c2**) We further conducted single-point calculations for each strand separately, and for the states with deleted central base. Next, the differences in energy and overall features between the duplex and these states were calculated. Note, that differences in IP, HOMO, and LUMO (*Δ*IP, *Δ*HOMO, and *Δ*LUMO) from varying molecular states do not have clear physical meaning and should be avoided from direct usage in machine learning. However, we include them in our dataset to enable the retrieval of the corresponding values for single-stranded and base-deleted states from the IP, HOMO, and LUMO of duplex states, i.e. by taking IP-*Δ*IP, HOMO-*Δ*HOMO and LUMO-*Δ*LUMO.

The schematic diagram of the generation of the dataset is shown in Fig. 2. The procedure is comprised of three stages: the building of the all-atom DNA models (a), geometry optimisation (b), and feature extraction with the corresponding single-point calculations (c). In the following subsections, we describe the details of each stage. The calculations were performed on the available Linux computing clusters hosted at the MRC Weatherall Institute of Molecular Medicine, University of Oxford (256 GB of RAM, dual Intel Xeon E5-2680v3 CPUs with 24 physical cores per node), and on our laboratory workstation (512 GB of RAM, Intel Xeon W-2295 CPUs with 18 physical cores). The dataset can be accessed through our GitHub page at https://github.com/SahakyanLab/DNAkmerQM or from Zenodo^33^. The R programming language^34^ was used as a front-end programming language in this work, and the code to generate the dataset can be retrieved from https://github.com/SahakyanLab/NucleicAcidsQM.

### All-atom model building for 7-mer DNA

As the maximum context span and the baseline in this work, the heptameric range of DNA was considered due to its known major influence on nucleotide and derivative properties^21^. However, our dataset also includes an analogue for the lesser, hexameric context, mainly generated for the use cases when an even-numbered range is needed for modelling. The DNA structures for all the k-mer sequence permutations in their B, A and Z conformations (Fig. 1) were generated by using the Nucleic Acid Builder (NAB) suit of programmes^35^ (Fig. 2a). NAB provides a function that replaces base pairs of a given template structure with any desired base pair, without altering the geometries of the backbone and sugar moieties. As templates for B, A and Z conformations of double-stranded DNA, representative X-ray crystallographic structures^36^ were used as adapted from the PDB files provided in WEB-3DNA^37^ (Fig. 2a). The end moieties for the 1^*st*^ and 7^*th*^ positions in the DNA models were capped by hydrogen atoms (at the O5′ and O3′ positions of deoxyribose for the 5′ and 3′ ends respectively). The total number of all the permutations for 7-mer sequences of four bases is 4^7^ = 16,384. However, considering the strand symmetry of double-stranded DNA, we can reduce this number, since, for instance, the sequence 5′-AAAAATT-3′ has 5′-AATTTTT-3′ as a complementary strand, hence the double-stranded DNA model for 5′-AAAAATT-3′ is the same for 5′-AATTTTT-3′ as well. We therefore generated 8,192 DNA models for each B, A and Z conformations, resulting in a total of 24,576 DNA models for heptamers. On average, one DNA model in our generated set contains 443 atoms (281 heavy, and 162 hydrogen atoms).

### Molecular mechanics optimisation of DNA

The above DNA structures were then geometry optimised *via* molecular mechanics (MM) force field, by using AmberTools21^38^ (Fig. 2b). The OL15 force field, specifically tuned and well tested for DNA^39^, was used. To account for the shielding of the negatively charged phosphate backbones, Born implicit solvation^40^ was used with the water environment dielectric constant defaulted to 78.5 in AmberTools21. We needed to relax the geometries in order to remove any tension and unrealistic arrangements upon NAB-driven base replacements. However, we still wanted to preserve the conformations in their desired original, B, A or Z, state. The electronic parameters provide a useful approximation rather than a definitive representation of all possible geometric states. As such, those serve as a foundational set of electronic parameters for the standard nucleic acid conformations, potentially emulating a wealth of derivative information useful for machine learning. We therefore had to pick an appropriate number for the allowed optimisation steps. Fig. S1a1 shows a relationship between root mean squared deviation (RMSD) of 5′-AAAAAAA-3′ B-DNA, from its original NAB-generated structure, and the number of optimisation steps. RMSD calculation was done using the Bio3D library^41^ in R, based only on non-hydrogen atoms. We found that RMSD converged within 5000 steps. Furthermore, no major conformational change or strand separation was observed in the structures before and after the convergence (Fig. S1a2). Figure S1a3 shows the RMSDs for all the heptamers of B-DNA. Note that the sequences are numbered lexicographically, that is, 5′-AAAAAAA-3′=1, 5′-AAAAAAC-3′=2, 5′-AAAAAAG-3′=3, and so on. The results show that the RMSD values for all modelled sequences are at around 1.0 Å (with an average of 0.72 and 0.03 standard deviation). The same is true for the A and Z conformations of DNA (see Fig. S1b,c).

### Semi-empirical quantum mechanics optimisation of DNA

We further optimised the DNA structures through quantum mechanics (QM) (Fig. 2b) by using the PM6-DH+ semi-empirical Hamiltonian under the restricted Hartree-Fock (RHF) approach, as implemented in MOPAC2016 programme^42^. PM6-DH+ with its correction for dispersion interactions, while benefiting from the relative low cost of the semi-empirical QM methods, has successfully reproduced electronic properties of many systems as accurately as the costly QM methods^43^. The water environment was accounted for through the intrinsic solvation with Conductor-like Screening Model (COSMO)^44^. The COSMO default 78.4 dielectric constant was used for water, as implemented in MOPAC2016. For the termination of QM optimisation, we used the energy gradient criterion in MOPAC, rather than limiting the optimisation steps. Fig. S2a1 shows the RMSD of 5′-AAAAAAA-3′ B-DNA from its initial state, as a function of energy gradient cutoff used to optimise the system and look at the structural snapshot. Similar to the MM case discussed above, we found that RMSD plateaus at around 1.0 Å even if a strict convergence criterion is applied. Fig. S2a2 shows B-DNA structure before and after optimisation until the energy gradient drops below 1.0 kcal/(mol ⋅ Å). No substantial conformational change, such as separation of strands, was observed upon such optimisation, keeping the structures within the designated B conformation. On the other hand, 10.0 kcal/(mol ⋅ Å) is recommended as the energy gradient criteria for large systems (http://openmopac.net/manual/gnorm.html), such as our heptameric double-stranded DNAs. Therefore, we adopted the maximum gradient of 10.0 kcal/(mol ⋅ Å) as our convergence criterion for the QM geometry optimisation. Similar to the MM case, compliance to low RMSD was observed for all our DNA sequences in their B (Fig. S2a3, with an average of 1.00 Å and 0.13 standard deviation). The same was true for A and Z (Fig. S2b,c) conformations as well.

### Feature calculation and extraction

We next extracted the electronic and structural features from the obtained refined DNA structures (Fig. 2c1). For the electronic features, we conducted further single-point QM calculations on the optimised duplex B-, A- and Z-DNA structures. We used the same PM6-DH+ (RHF) with COSMO solvation, but with additional keywords to request more detailed outputs and a full listing of electronic parameters. From these calculations, as general features for each DNA model, heat of formation (Ehof), ionisation potential (IP), dipole moment, highest occupied molecular orbital (HOMO) energy, and lowest unoccupied molecular orbital (LUMO) energy were extracted. We also extracted Mulliken charges and populations for the constituent atoms. Since treating charges for all atoms is not realistic for many machine learning setups, we calculated the summarised maximum, minimum, and mean values of the charges and population density values for each of the base, sugar, and phosphate moieties from 1^*st*^ to 7^*th*^ nucleotides at both  + and  − strands. In the same manner, we extracted electrostatic potential fitted (ESP) charges and populations^45^ and calculated the maximum, minimum, and mean values. Geometric parameters were calculated *via* Curves+ software^46^, upon which inter- and intra-strand parameters were extracted for the base pair arrangements and backbone angles respectively. Further features were obtained by conducting additional single-point calculations for each strand separately, by masking the other strand in the optimised duplex B-, A- and Z-DNA structures (Fig. 2c2). The difference of Ehof between the duplex and the two separate single strands was calculated as a simple proxy for DNA hybridisation energy. We also considered the duplex state with the 4^*th*^ central base removed and replaced by a hydrogen cap. The single-point calculation was conducted after optimising only this hydrogen position, with a stricter 1.0 kcal/(mol ⋅ Å) maximum energy gradient for the convergence criterion. Then, we calculated the difference of Ehof between the complete duplex and the base-removed states, as illustrated in Fig. 2c2. This difference should be related to how the central base is stabilised, through the stacking and hydrogen bonding interactions, within the context of the whole DNA sequence.

### Overall calculation costs

For our 24,576 7-mer models, the MM optimisations utilised  ~650 hours of CPU time (on average 95.2 seconds per model). The QM optimisations took  ~11,052 CPU hours (averaging 1,618.9 seconds per model). For the subsequent single-point calculations, it took on average 74.1 seconds per model in CPU time. Since there were five such calculations per model, it took an overall 2,529 CPU hours. This amounted to 593 CPU days of calculations, which we were able to conduct within about 3 months by utilising up to six computing nodes.

## Data Records

### File description

Table 1 shows the summary of the dataset obtained *via* the above procedure. Our dataset is comprised of 7 deposited dataset files for each k-mer range. The units of features are described in parentheses. The file “energy.txt” includes the overall parameters for the double-helical DNA in its B, A and Z states, that is Ehof (kcal/mol), dipole moment (debye), HOMO and LUMO energies (eV), and IP (eV). The file “denergy.txt” includes differences of Ehof upon de-hybridisation of the DNA, and the central base removal, calculated for B, A and Z conformations (units are the same as in “energy.txt”). The file “Mullik_Charge.txt” includes the maximum, minimum, and mean Mulliken charge values (in e units, where e = +1.602177 × 10^−19^ C) at the base, sugar and phosphate moieties for each nucleotide position in B-, A- and Z-DNA. The file “Mullik_Density.txt” similarly includes the maximum, minimum, and mean values of the Mulliken population density (dimensionless). The files “ESP_Charge.txt” and “ESP_Density.txt” contain datasets for the electrostatic potential fitted charges and populations respectively (units are the same as Mulliken charge and population). The file “Curves.txt” includes intra- and inter-strand geometric parameters for our sequences at their B, A and Z states (Å and degree are the units of distance and angle). The described dataset files include all our sequences, one row of features per sequence, with the first column indicating the sequence in the lexicographic order.

**Table 1:** File names, contents, and the number of features of the generated and deposited dataset.

File name	Content	The number of features
energy.txt	Energy and overall parameters	15
denergy.txt	Difference of energy and overall parameters	36
Mullik_Charge.txt	Mulliken charges	378
Mullik_Density.txt	Mulliken populations	378
ESP_Charge.txt	ESP charges	378
ESP_Density.txt	ESP populations	378
Curves.txt	Geometric data	945

Table 2 shows our naming rules used to identify the features in the dataset files. Examples are described for (1) energy and difference of energy: B_ds.Ehof means heat of formation energy of duplex B-DNA. B_ds.dEhof_ds_ss means the difference of heat of formation energies between duplex B-DNA and its single-strand states. (2) As an example of charges and populations: here, the plus strand is the strand that has a given sequence, and the minus strand is the complementary strand. For example, when we consider the 5′-AAAAAAA-3′ sequence, the plus strand is the strand that has AAAAAAA nucleotides, while the minus strand is the complementary strand that has TTTTTTT nucleotides. By this rule, B_ds_strandPlus_4_phos_mean.MullikenCharge means the mean value of Mulliken charge of a phosphate part of the 4^*th*^ nucleotide in a plus strand of B-DNA. (3) An example of geometric parameters: B_ds_strandPlus_4.Curves_Xdisp means X displacement of a base of 4^*th*^ nucleotide in a plus strand of B-DNA. Note that the meaning of geometric parameters is well summarised in the 3DNA paper^47^ and Fig. S3 in this work.

**Table 2:** Explanations of the abbreviations for the feature names used in our dataset.

Abbreviation	Explanation
B_ds, A_ds, Z_ds	Duplex B-, A- and Z-DNA.
Ehof, Dipole, IP, Ehomo, Elumo	Heat of formation energy, Dipole, Ionization potential, HOMO, LUMO.
dEhof_ds_*x*, dIP_ds_*x*, dEhomo_ds_*x*, dElumo_ds_*x*	Difference of Ehof, IP, Ehomo, and Elumo between the duplex and *x* states. *x* = ss(single strand), del1(the centre base at the plus strand is removed), del2(the centre base at the minus strand is removed).
strandPlus_*x*	*x*-th nucleotide from 5’ edge of the strand that has a given sequence.
strandMinus_*x*	The complementary nucleotide of strandPlus_*x*.
phos_*x*, sugar_*x*, base_*x*	*x* values for phosphate, sugar, and base parts of a nucleotide. *x* = mean, max (maximum), min(minimum).
MullikCharge	Mulliken charge.
MullikDensity	Mulliken population.
ESPCharge	ESP charge.
ESPDensity	ESP population.
Curves_x	Geometric parameters *x* of DNA calculated by Curves+. *x* = Shear, Stretch, … (Intra base parameters), Shift, Slide, … (inter base parameters), and Alpha, Beta, … (backbone parameters). To_3 in the inter parameters means inter parameters toward the 3’ end side while from_5 means inter parameters from the 5’ end side.

## Technical Validation

### Accuracy

As a proof of concept and demonstration of a potential application of our developed dataset in an actual DNA sequence-based machine learning initiative, below we demonstrate the exclusive use of DNAkmerQM features in predicting context-dependent spontaneous mutation rate constants for A to C mutation *via* machine learning. Our dataset and the quality of the features inside reflect the state-of-the-art semi-empirical QM methodology that can still be applied for such a large molecular system. The software and packages we used, which are AmberTools21, MOPAC2016, R language, and so on, have a long history of developments and validations in their respective publications. Furthermore, in the above analyses, we did not find any strange behaviour such as outlier values. Instead, we found that the tendencies in the dataset are not so different from our conventional knowledge. For example, the tendencies of data for B- and A-DNA (right-handed) are similar but different from data for Z-DNA (left-handed).

### Applicability

To demonstrate the intended power of our dataset in an actual DNA sequence-based machine learning initiative, here we showcase the exclusive use of DNAkmerQM features in predicting context-dependent spontaneous mutation rate constants for A to C mutation *via* machine learning.

#### Construction of a dataset for machine learning

**Fig. 3:**
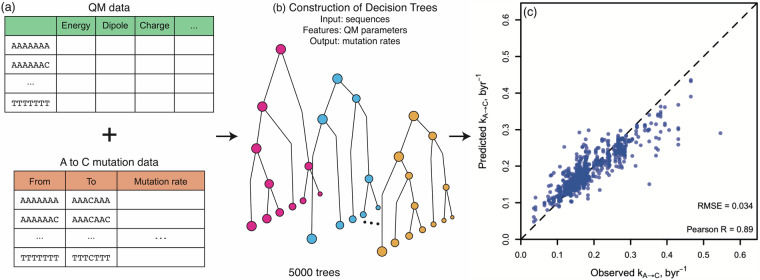
Developing a sequence-driven machine learning model for A to C mutation rates based solely on QM features mapped onto the sequence. Schematic illustrations are shown for (**a**) combining our QM dataset and an external dataset for A to C mutation rate constants (k_A→C_) and (**b**) constructing a machine learning model that predicts k_A→C_ from the sequence, based on the sequence-mapped QM features deposited in the DNAkmerQM dataset presented in this work. (**c**) The mutation rate constants predicted through our machine learning model *vs*. actual mutation rate constants (in byr^−1^, i.e. mutation per billion years for each such nucleotide site). The dashed line shows the diagonal for the ideal match.

The Trek (transposon exposed k-meric mutation rate constants) dataset provides comprehensive sequence-dependent mutation rates for the human genome, as obtained from LINE-1 remnants^21^. We combined our QM datasets, which contain features related to B-DNA in “energy.txt”, “denergy.txt”, and “Mullik_Charge.txt” with A to C mutation rate constants (k_A→C_) from the Trek dataset (Fig. 3a), resulting in 4096 samples with 102 features.

#### Development of a machine learning model

We divided this dataset into 80% for training (3279 samples) and 20% for pure test (817 samples). Next, we constructed tree-based Gradient Boosting Machine (GBM) models, by which decision trees are consecutively generated to predict the residual values of the ensemble of prior learner trees (Fig. 3b)^48–50^. GBMs are known to exhibit superior performance, often prevailing those of neural network-based models for tabulated data^51,52^. GBMs have flexible tunability by five hyperparameters (interaction depth, minimum child weight, bag fraction (sampling rate), learning rate, and the number of trees). These five hyperparameters are related to the overall architecture of the GBM model and drastically affect the performance. The development of GBMs thus involves a careful selection of the optimal combination of its hyperparameters to achieve the best performance. For this, a three-step procedure was employed in this study. (1) Construction of preliminary GBMs by a reasonable initial parameter set. (2) Feature reduction: GBMs provide the importance of features, that is, how much each feature contributes to the performance of GBMs. Based on the feature importance values of the preliminary GBM model, we excluded features that do not contribute to the model performance much. This drastically reduced the computation cost of the following procedure. (3) Grid search: after the reduction of features, we developed varying GBMs with various combinations of the five parameters. The performance for each model was measured by the root mean squared error (RMSE) from 10-fold cross-validation. We summarise the employed final hyperparameters (interaction depth = 11, the number of trees = 5000, learning late = 0.01, bag fraction = 0.8, and minimum child weight = 5), along with all the sampled ranges, in Table S1.

#### Validation of the machine learning model

From the production level GBM model obtained through the above procedure, we predicted A to C mutation rates from the pure test set. Fig. 3c shows a scatter plot between true values in the pure test set and the predicted values by our GBM model. The predicted mutation rate constants agreed very well with the actual values (Pearson’s R = 0.89, RMSE = 0.034). This thus demonstrates the potential of our dataset to provide a wealth of physicochemical features, which, even while used as sole features, are capable of generating a sophisticated machine learning model for a range of DNA sequence-based biological phenomena.

## Usage Notes

Since the 1^*st*^ and the 7^*th*^ nucleotides are located at the edge of the DNA segments used in the modelling, the usage of their features should be preferentially avoided if used in machine learning. For example, our machine learning model mentioned above shows a lower performance when we include these edge nucleotide data as features. Differences in IP, HOMO, and LUMO (*Δ*IP, *Δ*HOMO, and *Δ*LUMO in “denergy.txt”) do not have clear physical meanings for varying (not same) molecular systems, and thus these features should be avoided too. However, we include them in the dataset for the estimation of IP, HOMO, and LUMO for single strands and base-deleted states as detailed in the caption of Fig. 2.

### Supplementary information

Supplementary Information

## Data Availability

The dataset is publicly available on GitHub (https://github.com/SahakyanLab/DNAkmerQM) and Zenodo^33^ under the CC-BY license. The required code to generate the dataset is freely accessible under the CC-BY license from (https://github.com/SahakyanLab/NucleicAcidsQM).

## Code availability

All software and packages used in this study are freely distributed and available through their citations brought in the text.
