# Supplementary material for: Quantum mechanical electronic and geometric parameters for DNA k-mers as features for machine learning
Source: Sci Data. 2024 Aug 22;11:911. doi: 10.1038/s41597-024-03772-5 (PMC11341866; doi:10.1038/s41597-024-03772-5)
Supplement: Supplementary file 1 — Supplementary Information [file 41597_2024_3772_MOESM1_ESM.pdf]

# Supplementary Information
# Quantum mechanical electronic and geometric parameters for DNA k-mers as features for machine learning

Kairi Masuda, Adib A. Abdullah, Patrick Pflughaupt, and Aleksandr B. Sahakyan*

*MRC WIMM Centre for Computational Biology, MRC Weatherall Institute of Molecular Medicine, Radcliffe Department of Medicine, University of Oxford, Oxford, OX3 9DS, United Kingdom*

E-mail: aleksandr.sahakyan@imm.ox.ac.uk

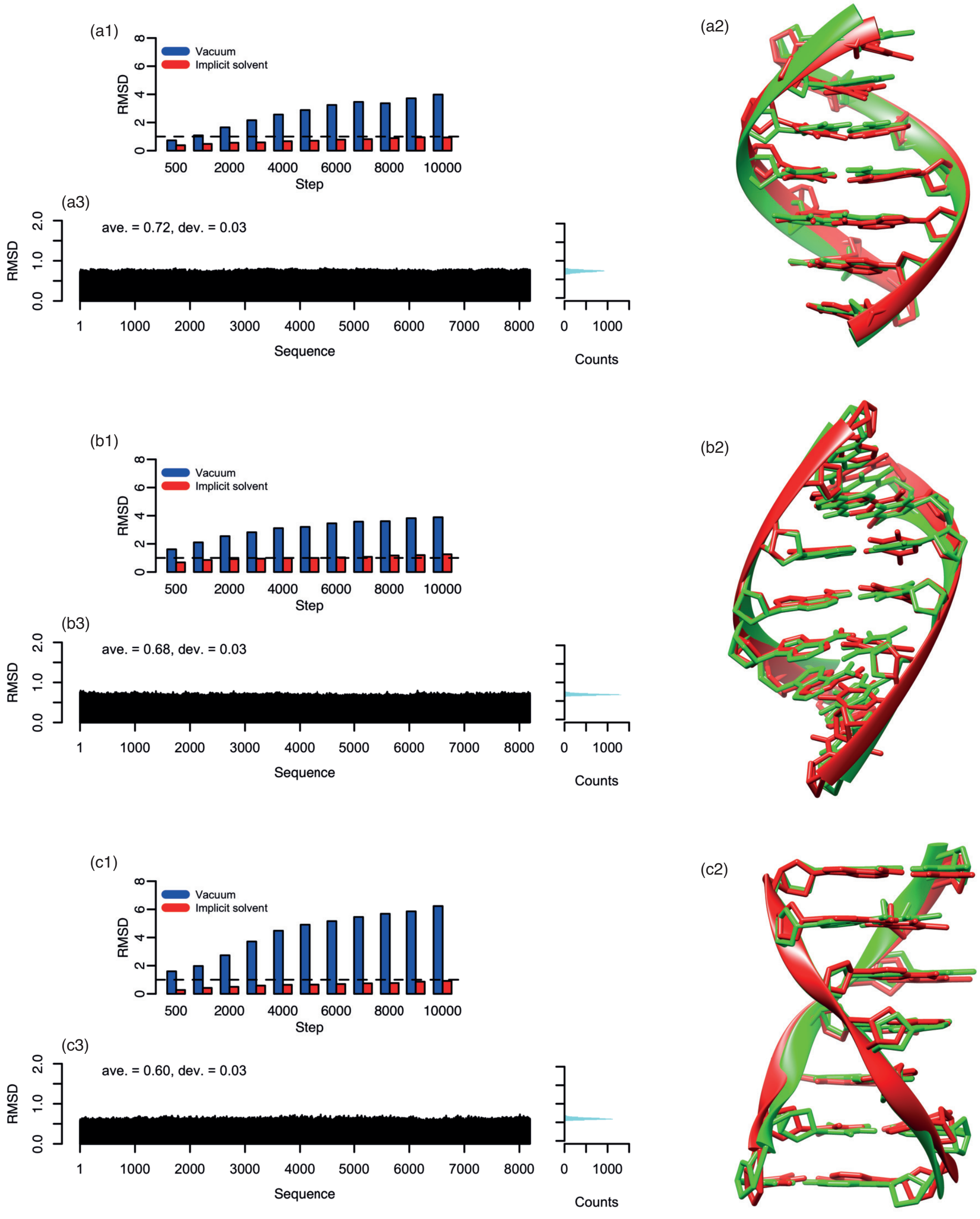


**Figure S1.** (a1) RMSDs of 5'-AAAAAAA-3' B-DNA as a function of MM geometry optimisation steps. Red bars indicate RMSDs of the B-DNA in water. For comparison, the RMSD of the B-DNA in a vacuum is shown by blue bars. (a2) 5'-AAAAAAA-3' B-DNA structures before (green) and after (red) 5000 optimisation steps. (a3) RMSDs of all 7-mers, 8192 sequences, of B-DNA after 5000 steps of geometry optimisation. Note that numbers were applied to each sequence in a dictionary order. That is, 5'-AAAAAAA-3' = 1, 5'-AAAAAAC-3' = 2, 5'-AAAAAAG-3' = 3, ... are indicated. The histogram is attached to the right. The same results for (b) A-DNA and (c) Z-DNA.

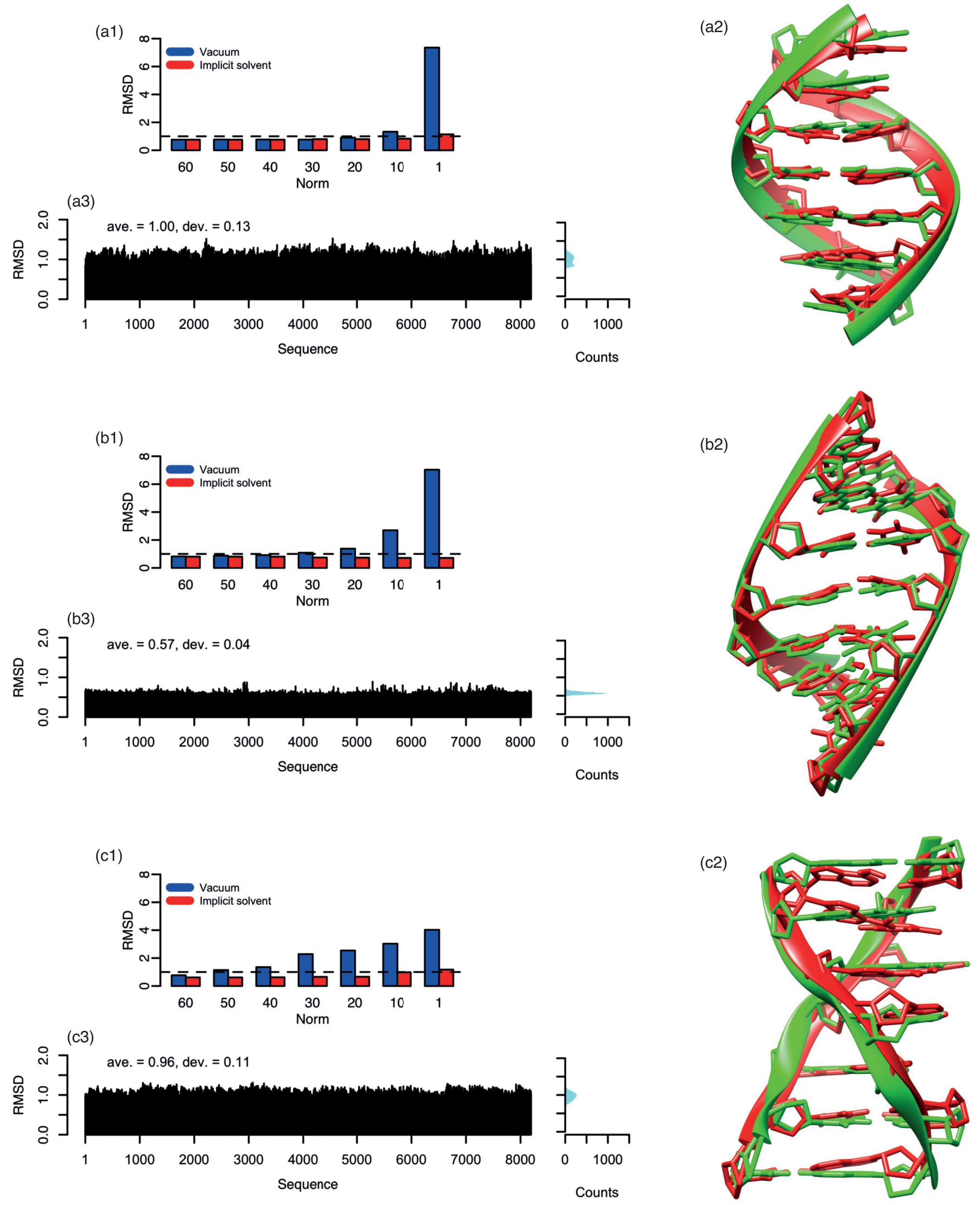


**Figure S2.** (a1) RMSDs of 5'-AAAAAAA-3' B-DNA after QM optimisation until an energy gradient norm becomes below a given value. (a2) 5'-AAAAAAA-3' B-DNA structures before (green) and after (red) optimisation until the corresponding energy gradient norm drops below 1 kcal/(mol·Å). (a3) RMSDs of all 7-mers, 8192 sequences, of B-DNA after optimisation until an energy gradient norm becomes below 1 kcal/(mol·Å). Note that numbers were applied to each sequence in a dictionary order. That is, 5'-AAAAAAA-3' = 1, 5'-AAAAAAC-3' = 2, 5'-AAAAAAG-3' = 3, ... are indicated. The histogram is attached to the right. Same results for (b) A-DNA and (c) Z-DNA.

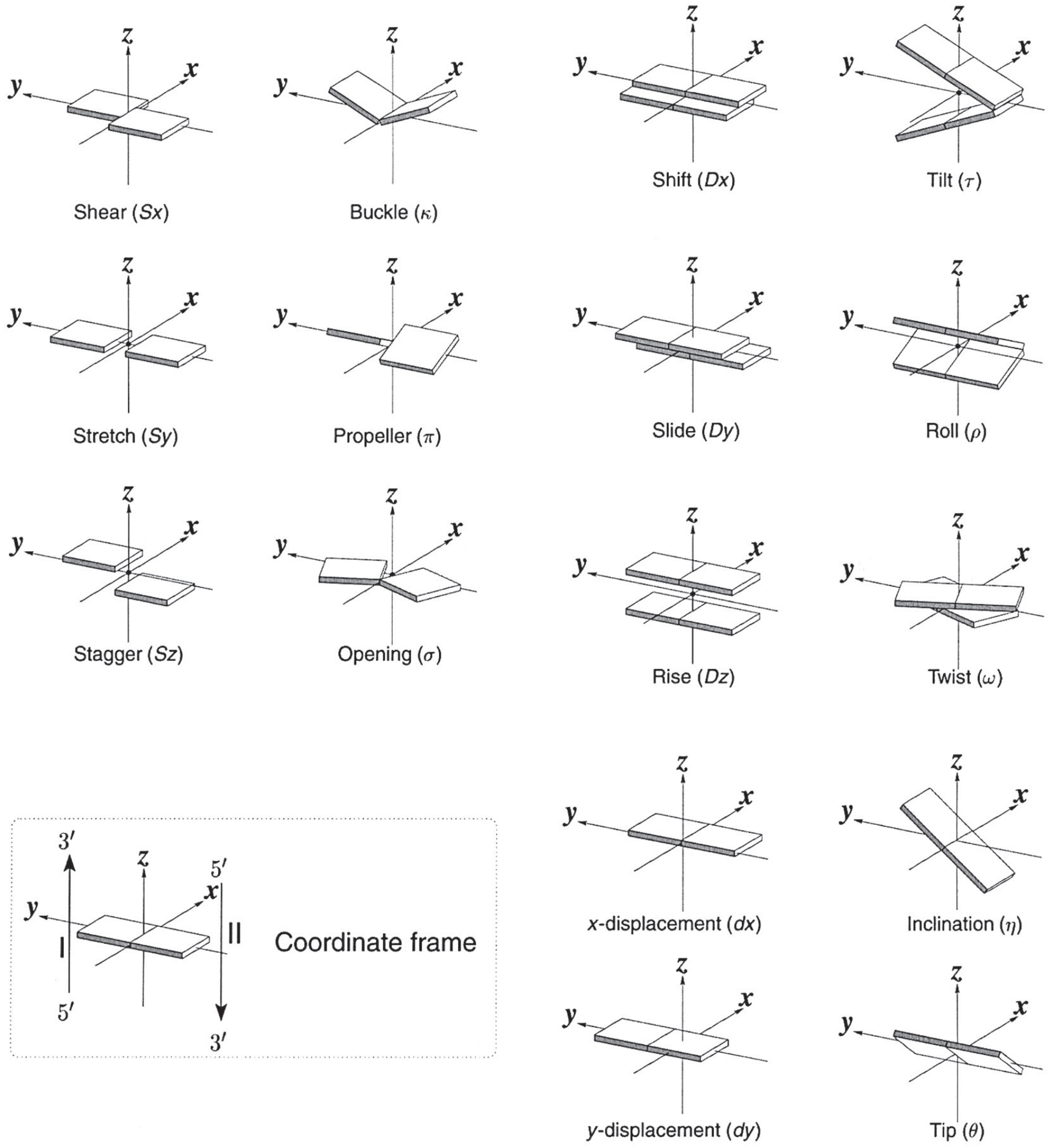


**Figure S3.** Definitions of the mechanical parameters taken from 3DNA paper (Lu, X. J. and Olson, *Nat.Protoc.* **3**, 1213–1227 (2008))

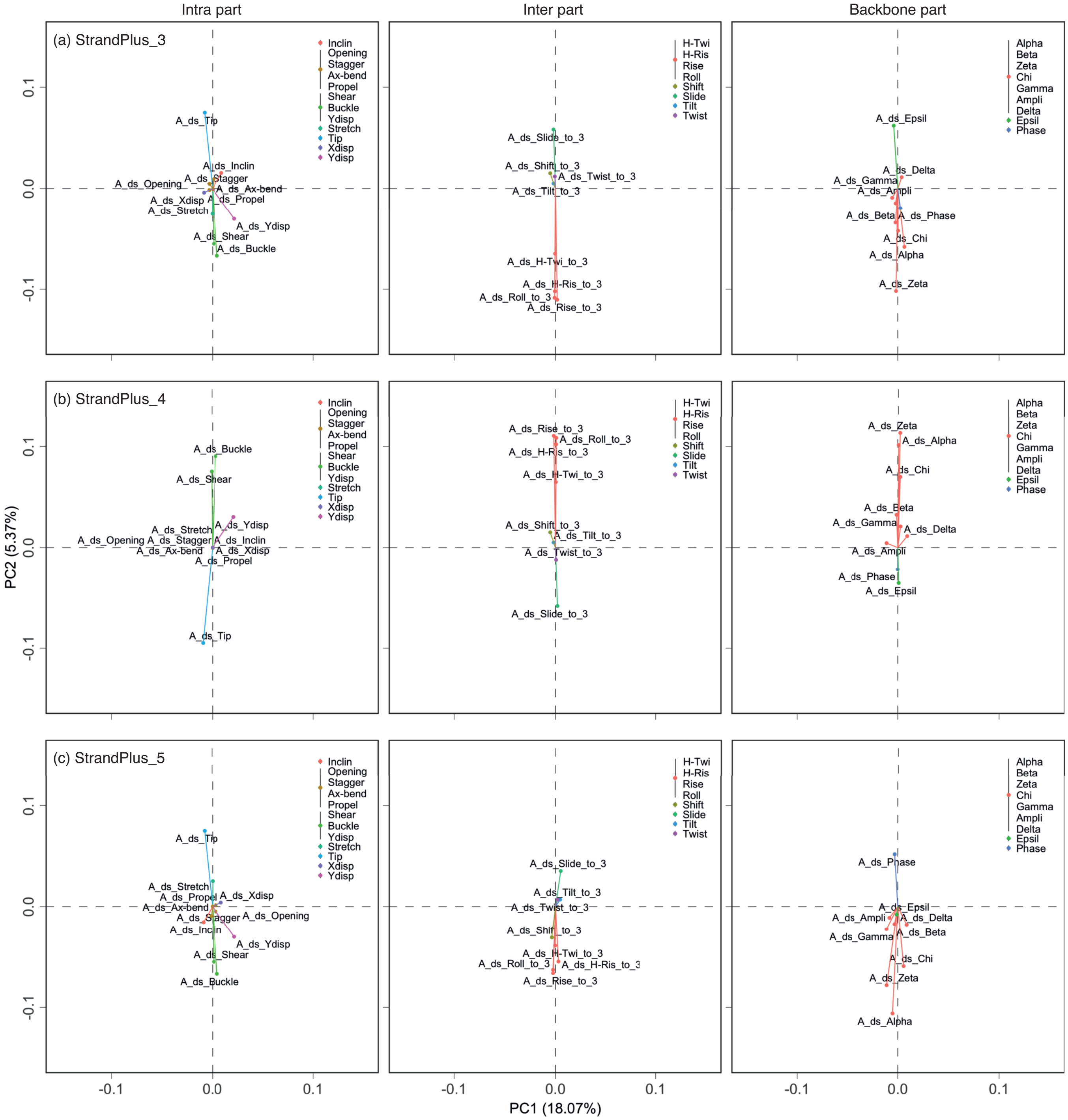


**Figure S4.** Loading plots by PCA of only mechanical features of A-DNA. Each figure corresponds to the intra, inter, and backbone parts at the nucleotide units of (a) strandPlue_3, (b) strandPlue_4, and (c) strandPlue_5 sites. To clearly show tendencies, features that show similar behaviours through strandPlue_3∼5 are grouped by colours.

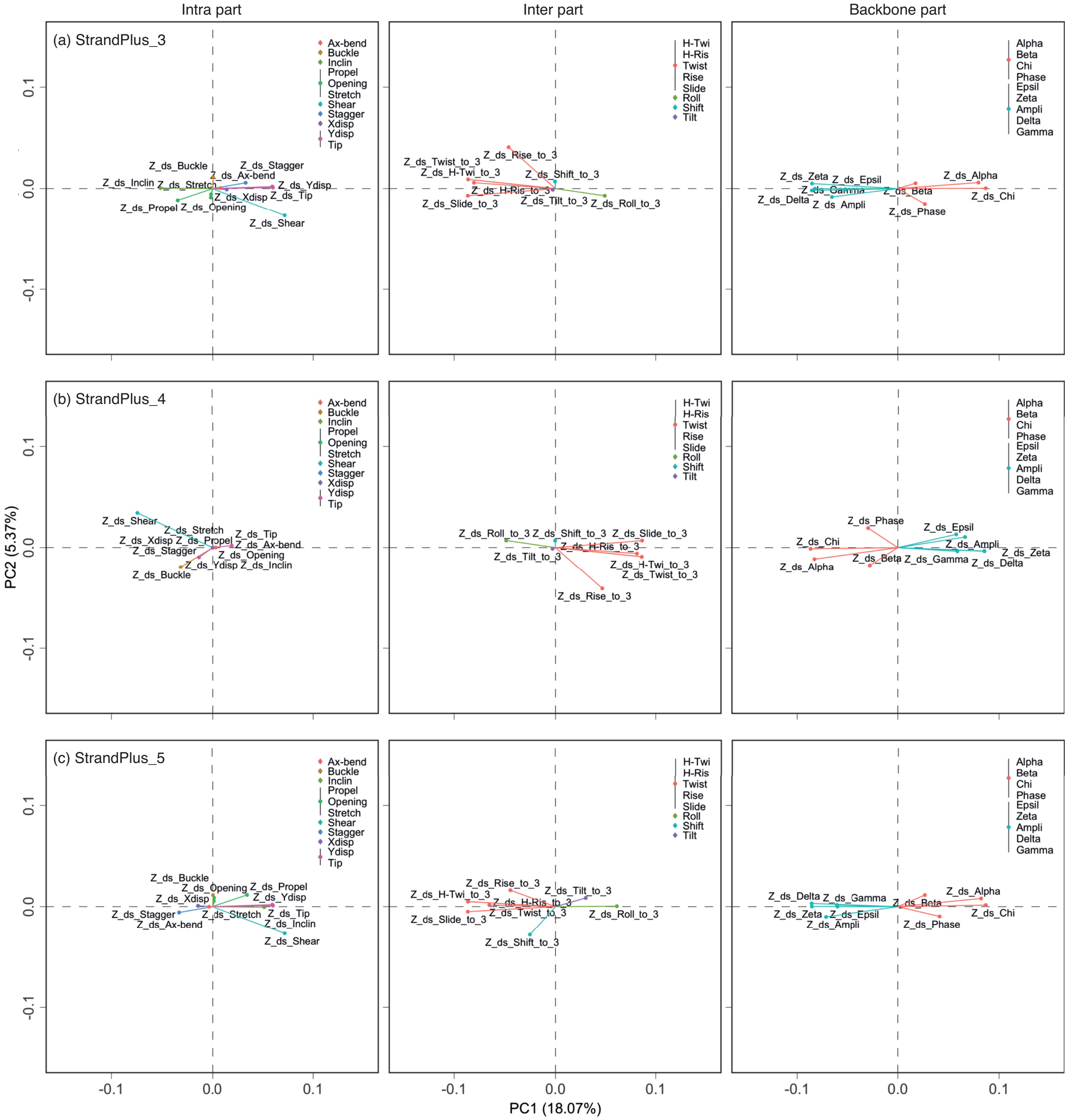


**Figure S5.** Loading plots by PCA of only mechanical features of Z-DNA. Each figure corresponds to the intra, inter, and backbone parts at the nucleotide units of (a) strandPlue_3, (b) strandPlue_4, and (c) strandPlue_5 sites. To clearly show tendencies, features that show similar behaviours through strandPlue_3∼5 are grouped by colours.

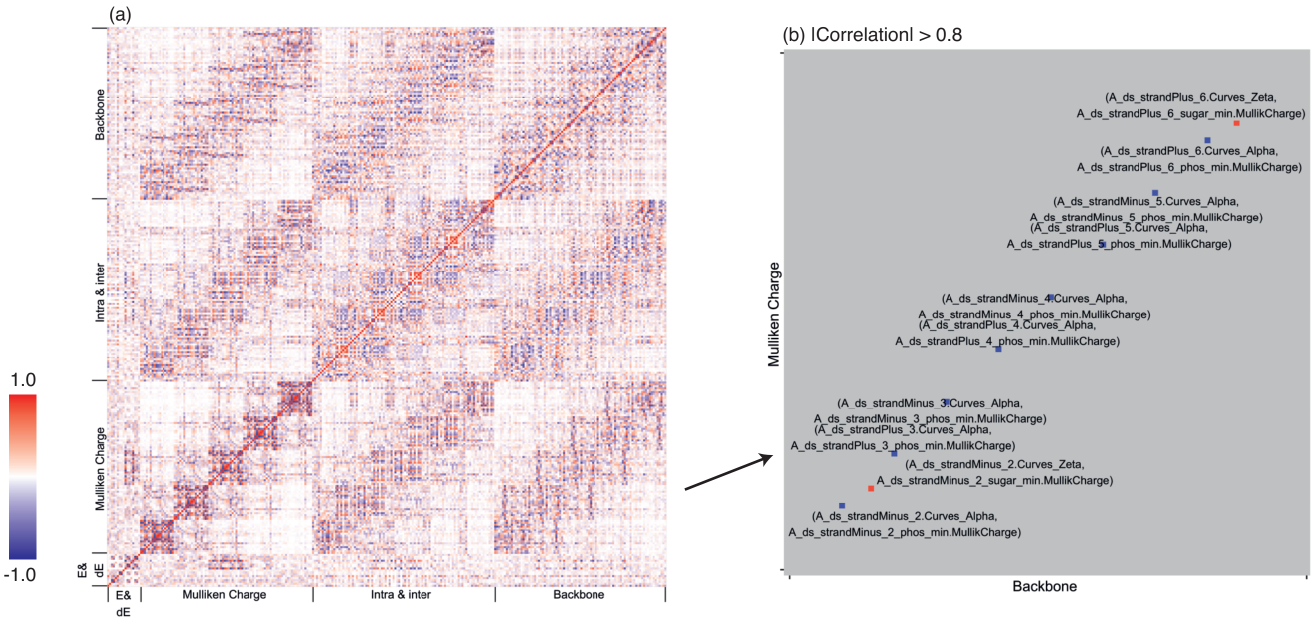


**Figure S6.** (a) A heat map of features related to A-DNA. For clarity, categories of features are shown instead of showing all features. The colour contour indicates a positive and negative correlation between features. (b) A heat map at the Backbone and Mulliken Charge regions (squares) with only high correlation points being shown.

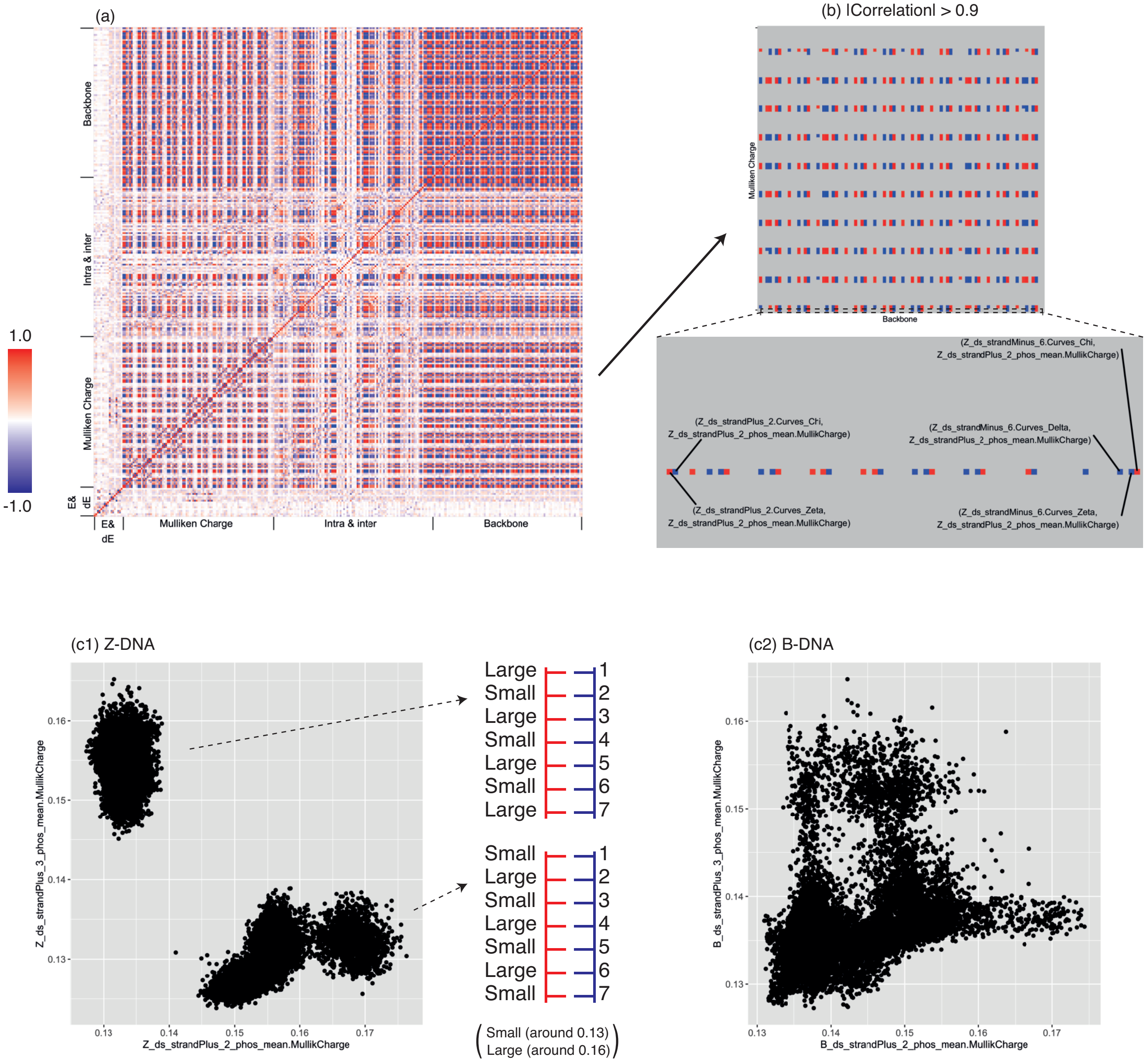


**Figure S7.** (a) A heat map of features related to Z-DNA. For clarity, categories of features are shown instead of showing all features. Colour contour indicates a positive and negative correlation between features. (b) A heat map at the Backbone and Mulliken Charge square with only high correlation points being shown. A row of Z_ds_strandPlus_2_phos_mean.MullikCharge is enlarged for clarity. (c) The mean values of Mulliken charge of the phosphate parts at the $2^{nd}$ and $3^{rd}$ nucleotides are plotted for Z-DNA and B-DNA. For Z-DNA, schematic images of pseudo-quantised/categorical charge states are shown.

**Table S1.** Hyperparameters for tuning the GBM for A→C mutation rate constants. Values for the architecture that shows the best performance is highlighted in red colour. In the feature selection stage, we first generated a model using all features (same as the preliminary model), then modelled using features that have importance > 1 (removing the features that are 100 or more times weaker than the most influential feature in the preliminary model), > 2, > 3, and > 4.

| | |
|---|---|
| Preliminary GBM model | |
| Number of features | 102 |
| Number of samples | 3279 |
| Performance metric | RMSE from 10-fold CV |
| Interaction depth | 6 |
| Minimum child weight | 5 |
| Bag fraction | 1 |
| Learning rate | 0.01 |
| Number of trees | {500, 750, 1000, 1500, 2000, 2500, 3000, 3500} |
| Best performance | 0.0423 |
| Feature selection | |
| Number of features | 102, 75, 47, 35, 29 (Importance>0,1,2,3,4) |
| Number of samples | 3279 |
| Performance metric | RMSE from 10-fold CV |
| Interaction depth | 6 |
| Minimum child weight | 5 |
| Bag fraction | 1 |
| Learning rate | 0.01 |
| Number of trees | {500, 750, 1000, 1500, 2000, 2500, 3000, 3500} |
| Best performance | 0.0387 |
| Rough grid search | |
| Number of features | 29 |
| Number of samples | 3279 |
| Performance metric | RMSE from 10-fold CV |
| Interaction depth | {6, 9, 12} |
| Minimum child weight | {1, 5, 25} |
| Bag fraction | {0.6, 1} |
| Learning rate | {0.01, 0.05} |
| Number of trees | {500, 750, 1000, 1500, 2000, 2500, 3000, 3500} |
| Best performance | 0.0358 |
| Fine grid search | |
| Number of features | 29 |
| Number of samples | 3279 |
| Performance metric | RMSE from 10-fold CV |
| Interaction depth | {11, 12, 14} |
| Minimum child weight | {5, 10 15} |
| Bag fraction | {0.8, 1.0} |
| Learning rate | 0.01 |
| Number of trees | {1500, 2000, 2500, 3000, 3500, 4000, 4500, 5000} |
| Best performance | 0.0354 |
